# Changes in the blood metabolome of Wagyu crossbred steers with time in the feedlot and relationships with marbling

**DOI:** 10.1038/s41598-020-76101-6

**Published:** 2020-11-04

**Authors:** Samantha Connolly, Anthony Dona, Darren Hamblin, Michael J. D’Occhio, Luciano A. González

**Affiliations:** 1 Sydney Institute of Agriculture, The University of Sydney, Sydney, NSW 2006 Australia; 2 School of Life and Environmental Sciences, Faculty of Science, The University of Sydney, Camden, NSW 2570 Australia; 3 Hamblin Pty Ltd, ‘Strathdale’, Blue Mountain, Sarina, QLD 4737 Australia; 4 Kolling Institute of Medical Research, Northern Medical School, The University of Sydney, St Leonard’s, NSW 2065 Australia

**Keywords:** Computational biology and bioinformatics, High-throughput screening, Metabolomics, Metabolomics, Animal physiology

## Abstract

Wagyu crossbred steers (n = 167) were used to (1) compare the metabolome of individual animals at two distant time-points (days 196 and 432) in a feedlot (this corresponded to 272 and 36 days before slaughter); and (2) determine relationships between the metabolome and marbling, and the effect of days in the feedlot (time-points) on these relationships. ^1^H NMR spectroscopy followed by standard recoupling of variables analysis produced 290 features or ‘peaks’ from which 38 metabolites were identified. There was a positive correlation between the relative concentration (RC) at days 196 and 432 for 35 of 38 metabolites (P > 0.05). The RC of 21 metabolites mostly involved in muscle energy and glucose metabolism increased (P < 0.05) from day 196 to 432, and the RC of 13 metabolites mostly involved in lipid metabolism decreased (P < 0.05). There were 14 metabolites correlated with marbling including metabolites involved in energy and fat metabolism (glucose, propionate, 3-hydroxybutyrate, lipids). The relationship between marbling and the RC of metabolites was affected by time-point, being positive for 3-hydroxybutyrate and acetate (P < 0.05) at day 432 but not at day 196. The findings indicate that the blood metabolome in Wagyu crossbred steers changes with time in a feedlot. Notwithstanding, the metabolome has potential to predict marbling in Wagyu. The ability to predict marbling from the blood metabolome appears to be influenced by days in a feedlot and presumably the stage of development towards a mature body conformation.

## Introduction

Cattle with Wagyu (*Bos taurus*) genetics have a high propensity to accumulate intramuscular fat (marbling) and are targeted at markets for premium beef^1,2^. Wagyu and Wagyu crossbred cattle typically undergo periods of 350–650 days in a feedlot to achieve high marbling. Animals that fail to achieve the necessary marbling are heavily discounted, and the cost of production can be greater than the market value. The final grading of Wagyu carcasses occurs after slaughter, and hence there is considerable interest in identifying ways to predict the carcass outcome for individual animals. Marbling has a relatively high heritability (0.38–0.50)^2,3^ in Wagyu.

The blood metabolome has emerged as an important source of biomarkers that have the potential to predict production, health and disease in livestock^4–6^. In a recent study, the blood metabolome of Wagyu crossbred steers was found to be associated with important production traits such as growth rate, carcass weight, subcutaneous rump fat, and marbling (intramuscular fat)^7^. Certain metabolites were positively associated with different production traits and other metabolites had a negative association. Irrespective, the findings showed that the blood metabolome has potential to offer biomarkers that can be used to select individual Wagyu steers for performance in a feedlot. Sire of the steers was the single most important factor affecting the blood metabolome, which suggests that metabolome biomarkers could potentially also be used to select Wagyu sires^7^.

Further, in the aforementioned study with Wagyu steers, the blood metabolome was determined 300–400 days before animals were slaughtered^7^. Cattle undergo metabolic and physiological adjustments during growth and development towards a mature body conformation and size. Bone growth is fastest in younger animals and precedes muscle growth^8,9^. Fat deposition increases with age and as animals reach a mature body size. Amongst the different fat deposits, abdominal fat is the earliest to accumulate followed by intermuscular fat, subcutaneous fat, and then marbling^1^. Based on the results within the present study, features of the blood metabolome in cattle change significantly with time in a feedlot. It is entirely feasible that specific associations of the metabolome with production and carcass traits may also differ over time. For example, it could be predicted that metabolites linked to marbling may have a stronger association with marbling in older, maturing animals.

With the above background in mind, the aims in the present study were (1) to compare and contrast the metabolome for individual Wagyu crossbred steers at days 196 and 432 in a feedlot (this corresponded to 272 and 36 days before slaughter); and (2) to determine relationships between the metabolome and marbling, and the effect of days in the feedlot (time-points) on these relationships. The sampling time-points were 236 days apart providing enough time for animals to differ in metabolic and physiological status related to degree of maturity. The current study also looked at identifying specific metabolites that might be used as predictive biomarkers of high value cattle. The hypothesis of the present study was that significant changes in the metabolome of cattle occur over time at the feedlot because of physiological maturity and fat deposition. Such changes would be reflected in greater influence of metabolites involved in intramuscular fat deposition such as glucose, lipids, and their precursors later in the feeding period.

## Materials and methods

### Animals and experimental design

**Figure 1:**
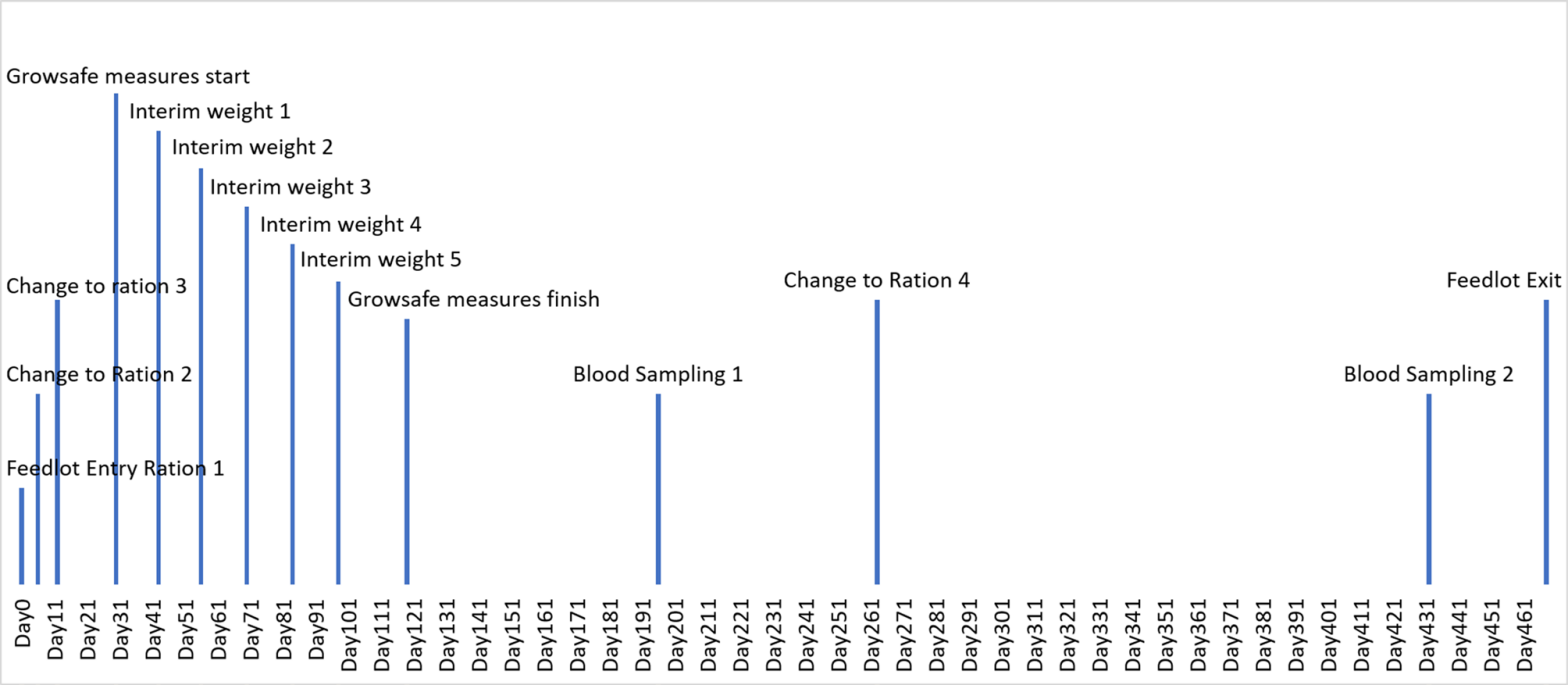
Timeline of events relative to experimental day for Wagyu crossbred feedlot cattle to measure blood metabolomics.

Wagyu crossbred steers (n = 167) from a single cohort and same management were used in the study. There was an even distribution of animals with different Wagyu genetics with 54, 56 and 53 animals of first cross (50% Wagyu), second cross (75% Wagyu) and third cross and above (≥ 87.5% Wagyu), respectively. Fullblood Wagyu bulls were predominantly crossed with Shorthorn (50%), Brahman (38%), Angus (10%), and other breeds (12%). Animals were maintained in a commercial feedlot in southern Queensland, Australia, and allowed ad-libitum consumption of feed. Changes in diet were implemented at days 6, 12 and 263 in the feedlot (Fig. 1). Diet composition and number of days each diet was fed are shown in Table 1 with chemical analysis done for diets 3 and 4 when blood sampling occurred. The animals entered an individual intake measurement system (GrowSafe, Calgary, Alberta, Canada) on day 30 with weights being measured every 14 days after entry for 90 days. Animals were sampled at day 196 (early feedlot period) and day 432 (late feedlot period) (Fig. 1). These sampling time-points corresponded to 272 and 36 days before animals were slaughtered (Fig. 1).

**Table 1:** Diet ingredients and composition.

Ingredient	Unit	Diet 1	Diet 2	Diet 3	Diet 4
Steam flaked barley	% as fed	21.5	28.5	37.5	47.0
Steam flaked wheat	% as fed	21.0	22.5	14.5	19.0
Finisher supplement	% as fed	5.0	5.2	0.0	1.7
Growth supplement	% as fed	0.0	0.0	5.0	3.5
Molasses	% as fed	12.0	10.1	4.8	5.0
Vegetable oil	% as fed	0.0	1.2	1.4	1.5
Brewers sweet grain	% as fed	0.0	0.0	15.0	8.0
Sunflower meal	% as fed	5.5	5.0	1.5	0.0
Corn silage	% as fed	12.0	12.0	12.8	9.8
Barley straw	% as fed	12.0	9.5	7.5	4.5
Lucerne hay	% as fed	11.0	6.0	0.0	0.0
**Chemical composition**					
Dry matter	%	–	–	94.8	95.2
Moisture	%	–	–	5.2	4.9
Acid detergent fibre	%	–	–	9.0	7.0
Dry matter digestibility	%	–	–	81.0	85.0
Inorganic ash	%	–	–	6.0	6.0
Organic matter	%	–	–	94.0	94.0
Crude fat	%	–	–	4.0	4.1
Crude protein	%	14.1^a^	13.8^a^	12.5	12.4
Neutral detergent fibre	% DM	29.3^a^	25.6^a^	20.0	17.0
Metabolizable energy	MJ/kg DM	10.9^a^	11.6^a^	13.0	13.5
Ionophore (monesin)	ppm	21.3^a^	22.2^a^	23.4^a^	23.9^a^
Net energy of gain	MJ/kg DM	4.4^a^	4.9^a^	5.5^a^	6.0^a^
Net energy of maintenance	MJ/kg DM	7.0^a^	7.6^a^	8.3^a^	8.9^a^

^a^Values are estimates from feed composition tables of the ingredients making up the diet.

Blood samples for metabolome analysis were obtained at days 196 and 432. On sampling days, animals were removed from their pens at 0600 h before feeding, and blood was collected between 0700 and 1030 h. An 18G needle and evacuated lithium heparin tube (Vacutainer BD, Becton Dickinson, Franklin Lakes, USA) were used to take coccygeal vein samples. Samples were immediately placed on ice for up to 20 min and centrifuged at 10,000 × *g* for 15 min. Plasma was then stored at − 80 °C until metabolome analysis.

### Carcass data

Carcass grading data was recorded by an accredited assessor using the Aus-Meat method^10^ with measurements of hot standard carcass weight (HSCW), marbling score, rib eye muscle area (EMA), and subcutaneous rib and rump fat thickness. The subjective Aus-Meat marbling score was assessed by a trained, accredited assessor on a scale of 0 to 9+ with 0 being the least and 9+ the greatest marbling. Marbling percentage was measured objectively using a hyperspectral camera (camera marbling, CM) (HK-333 camera; Hayasaka Rikoh Co. Ltd., Sapporo, Japan)^11^.

### Metabolite profiling

Plasma samples were prepared for metabolome analysis in accordance with a published protocol^12^. Samples were thawed at room temperature and an aliquot (350 μL) was mixed with 350 μL of aqueous (80% H_2_O:20% D_2_O) phosphate buffer solution including 0.075 M NaH_2_PO4, pH = 7.4 (KOH adjusted), 0.1% sodium azide, and 1 mM 3-141 trimethylsilyl-1-[2,2,3,3,-2H4] propionate (TSP) as an internal standard. Samples were mixed on a vortex for 30 s and centrifuged at 6,000 × *g* for 10 min. Aliquots (600 µL) of supernatant for each sample were pipetted into 5 mm NMR tubes for ^1^H NMR analysis (Bruker, SampleJet 5 mm, Billerica MA, USA). A quality control comprising equal volumes of approximately 10 samples prepared independently and were included every 15th sample in the NMR analysis.

A Bruker Advance III 600 MHz spectrometer equipped with a 5-mm TCI cryoprobe was used to analyze the samples. Samples were refrigerated at 4.85 °C prior to acquisition and run using a Sample Jet in automatic mode. Data was collected at 36.85 °C for 20 min. Noesygr and cpmgpr1d pulse sequences (32 scans collected for each experiment) were used to acquire the 1D ^1^HNMR spectra. Irradiation of the solvent (water) resonance was applied during pre-saturation delay (4.0 s) for all spectra and for the noesy also during the mixing time (0.01 s). Several of the pulse sequence parameters were optimized for each sample, particularly the 90° pulse (~ 12 μs). The data was collected for each sample with approximately 32 k (cpmg) or 96 k (noesy) real data points and processed with an exponential line broadening of 0.3 Hz prior to Fourier transformation.

Spectral data was imported into Matlab 7.0 software (Mathworks, Narick, MA). Each individual spectra was aligned and automatically phased, baseline corrected and referenced to the α-C_1_H-Glucose doublet at 5.233 ppm^13^. Spectra was then normalized using probabilistic quotient normalization. Statistical recoupling of variables (SRV) was used on the processed spectrum to calculate the start and endpoint of spectral components or clusters according to Blaise et al.^14^. The SRV output contains the clusters or peaks with the area under the curve calculated which is equivalent to the relative concentration (RC) of each individual peak. Sample spectra were then imported into Chenomx NMR Suite Professional (Chenomx Inc., Edmonton, AB, Canada), which was used as the reference library to identify peaks or features that belong to a metabolite according to its ppm using the spectral library along with published literature^15^. The RC of all peaks that belonged to the same metabolite were added to obtain the total RC of each metabolite for analysis.

### Statistical analyses

The spectral features relative concentration was multiplied by a factor of 10^6^ to reduce the number of decimal places. All statistical analyses were performed using SAS (v 9.4; SAS Institute Inc., Cary, NJ, USA). Pearson correlation coefficients were calculated between the RC of the identified metabolites at days 196 and 432 to determine the relationship between them across animals. Principal component analysis (PCA) was conducted using 38 identified metabolites to reduce the dimensionality of the dataset and then examine the effect of sampling time-point on the clustering and separation along the principal components (PC). Components with an eigenvalue > 1 were used in the final PCA and data is presented for the first three PC explaining the largest variation in the dataset. A generalized linear model (GLM) was used on the PCA scores output to examine the effect of time-point and Pearson correlation coefficients between marbling and the PC scores were also calculated.

The differences between time-points in the RC of metabolites were analyzed using a mixed-effects linear regression model containing the fixed effect of camera marbling (CM) as a covariate, time-point as the repeated measure, and the CM × time-point interaction. This model tested for different slopes between time-points, i.e. the regression between CM and the dependent variable for each time-point. Animal ID was a random effect. Any factor which was not significant was removed from the model and the model was re-run. All data was checked for normality and log-transformed where required. Outliers were detected using studentized residual and those strong residuals with a value > 3.5 or < − 3.5 were removed from the dataset. There were 50 outliers in total that were removed out of 21,759 data points analyzed.

### Animal ethics

The protocol of the present study was approved by the institutional animal ethics committee of The University of Sydney (approval #1125). The study was undertaken in accordance with the Australian code for the care and use of animals for scientific purposes 8th Edition 2013.

## Results

### Carcass attributes

Descriptive statistics of carcass measurements, weights and feed intake are shown in Table 2. Feedlot exit weight had greater variation than feedlot induction weight. The average marbling score was 6.66 and average camera marbling (CM) was 27.81%; however, these values showed wide ranges. Marbling score had greater variability compared to CM. Carcass weight and eye muscle area (EMA; *Longissimus Thoracis et Lumborum,* LTL) had the lowest coefficients of variation (CV) of all measurements. Feed intake in relation to percentage of body weight while in the Growsafe system had an average of 2.86% of BW and coefficient of variation of 10.25%.

**Table 2:** Descriptive statistics of Wagyu crossbred steers (n = 167).

Variable	Minimum	Mean	Maximum	Standard error	Coefficient of variation
Wagyu percent (%)	50.0	71.7	98.7	0.99	24.87
Age at induction (days)	460	614	1041	6.89	20.14
Feedlot induction weight (kg)	246	332.0	430	1.63	8.81
Feedlot exit weight (kg)	554	731.4	929	3.96	9.55
ADG feedlot (kg)	0.42	0.87	1.30	0.008	17.34
Aus-meat marble score	2.00	6.66	9.00	0.112	10.89
Camera marbling (%)	16.3	27.81	44.5	0.325	13.82
HSCW (kg)	323	427	542	2.253	6.40
EMA (cm^2^)	60	79.4	98	0.469	1.87
Rump fat (mm)	10	16.4	37	0.301	29.84
Rib fat (mm)	3	7.10	22	0.172	24.67
Feed intake (% body weight)^a^	2.05	2.86	3.61	0.016	10.25

^a^Feed intake measured using the Growsafe electronic feeders from 30 to 120 days on feed.

### Metabolome spectral data

**Figure 2:**
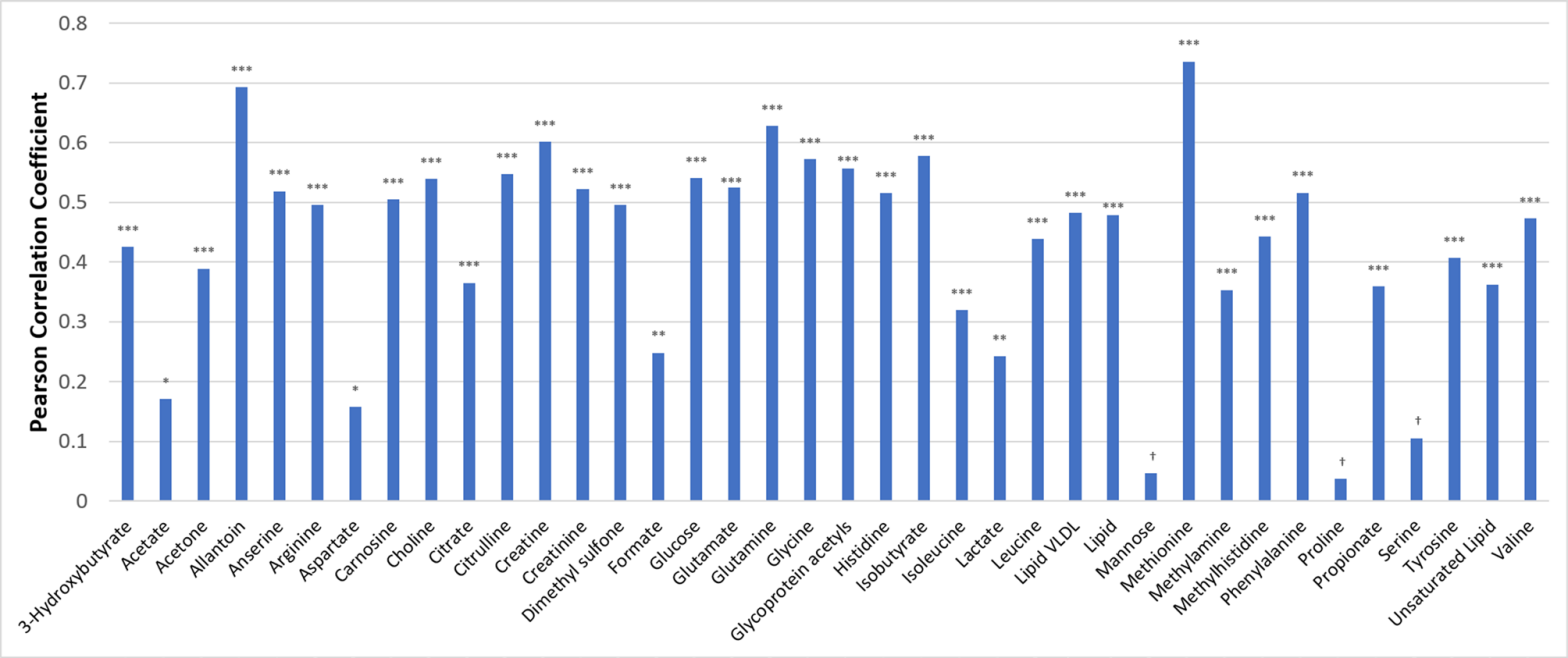
Pearson correlation coefficient between the relative concentration of plasma metabolites at days 196 and 432 in a feedlot in Wagyu crossbred steers. ***, **, *, † is for P ≤ 0.001, P ≤ 0.01, P ≤ 0.05 and P ≤ 0.10, respectively.

The dataset produced from analysis of the metabolome spectral data using statistical recoupling of variables (SRV) contained 290 peaks or clusters which were mapped to the spectral library of Chenomx. This identified 38 metabolites based on the ppm of the individual clusters from the spectral library and published literature^15^. Pearson correlation coefficients between the RC of each feature and each metabolite at days 196 and 432 were calculated. The correlation between the RC at days 196 and 432 ranged from − 0.13 to + 0.78 across all 290 features (data not shown). None of the negative correlation coefficients were significantly different than zero (*P* > 0.05) and 215 of 290 features with r > + 0.15 were significant (*P* < 0.05). Of 38 identified metabolites, only serine, proline and mannose were not significant (*P* > 0.05) with the remaining 35 metabolites showing a positive correlation between the RC at days 196 and 432 (Fig. 2).

### Principal component analysis

**Figure 3:**
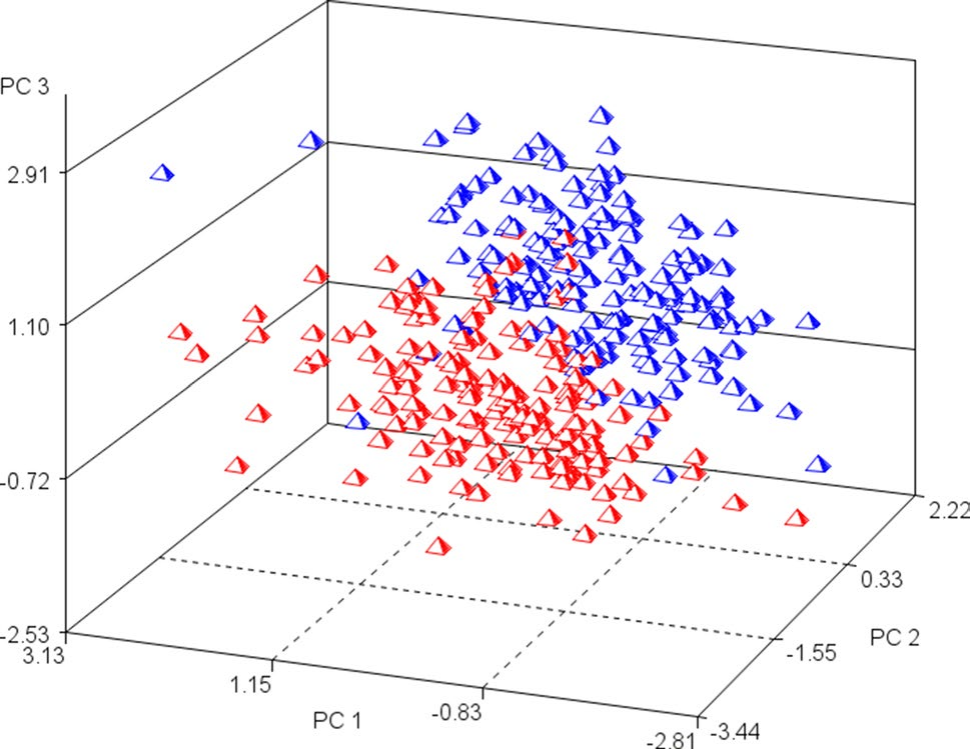
Score plot of the top three principal components (PC 1 to PC 3) obtained from 38 blood metabolites of Wagyu crossbred steers sampled at day 196 (blue) and day 432 (red) in a feedlot.

The principal component analysis (PCA) score plot indicated a clear separation between samples taken at day 196 compared with day 432, with the first three components explaining 61.22% of the variation in the dataset (Fig. 3). Further analyses of the principal component (PC) scores showed that most animals showed positive values for PC2 and PC3 on day 196 but negative PC2 and PC3 on day 432 (Table 3). PC1 and PC4 were negative at day 196 and positive at day 432, although the difference between time-points was smaller compared to PC2 and PC3 (Table 3).

**Table 3:** The effect of time-point in a feedlot on five principal components (PC) and Pearson correlation coefficients between principal component scores and marbling of Wagyu crossbred steers.

	Time-point		P-value	R^2^	Pearson r with marbling
	Day 196	Day 432			
PC1	− 0.279 ± 0.0758	0.277 ± 0.0756	< 0.001	0.077	0.110*
PC2	0.561 ± 0.0654	− 0.558 ± 0.0652	< 0.001	0.314	− 0.183***
PC3	0.517 ± 0.0676	− 0.514 ± 0.0674	< 0.001	0.267	0.161**
PC4	− 0.224 ± 0.0769	0.223 ± 0.0767	< 0.001	0.050	− 0.159**
PC5	0.021 ± 0.0789	− 0.021 ± 0.0787	0.708	0.0004	0.155**

*PC* principal component.

***, **, * P ≤ 0.001, P ≤ 0.01 and P ≤ 0.05, respectively, for the Pearson correlation coefficients.

The R^2^ values indicated that PC2 and PC3 were the two components that explained the largest proportion of the variability between time-points. All PC were significantly correlated with marbling (*P* < 0.05); however, PC2 had the largest Pearson coefficient. Both PC2 and PC4 were negatively correlated with marbling (*P* < 0.05) whereas PC1, PC3 and PC5 were positively correlated with marbling (*P* < 0.05; Table 3).

**Figure 4:**
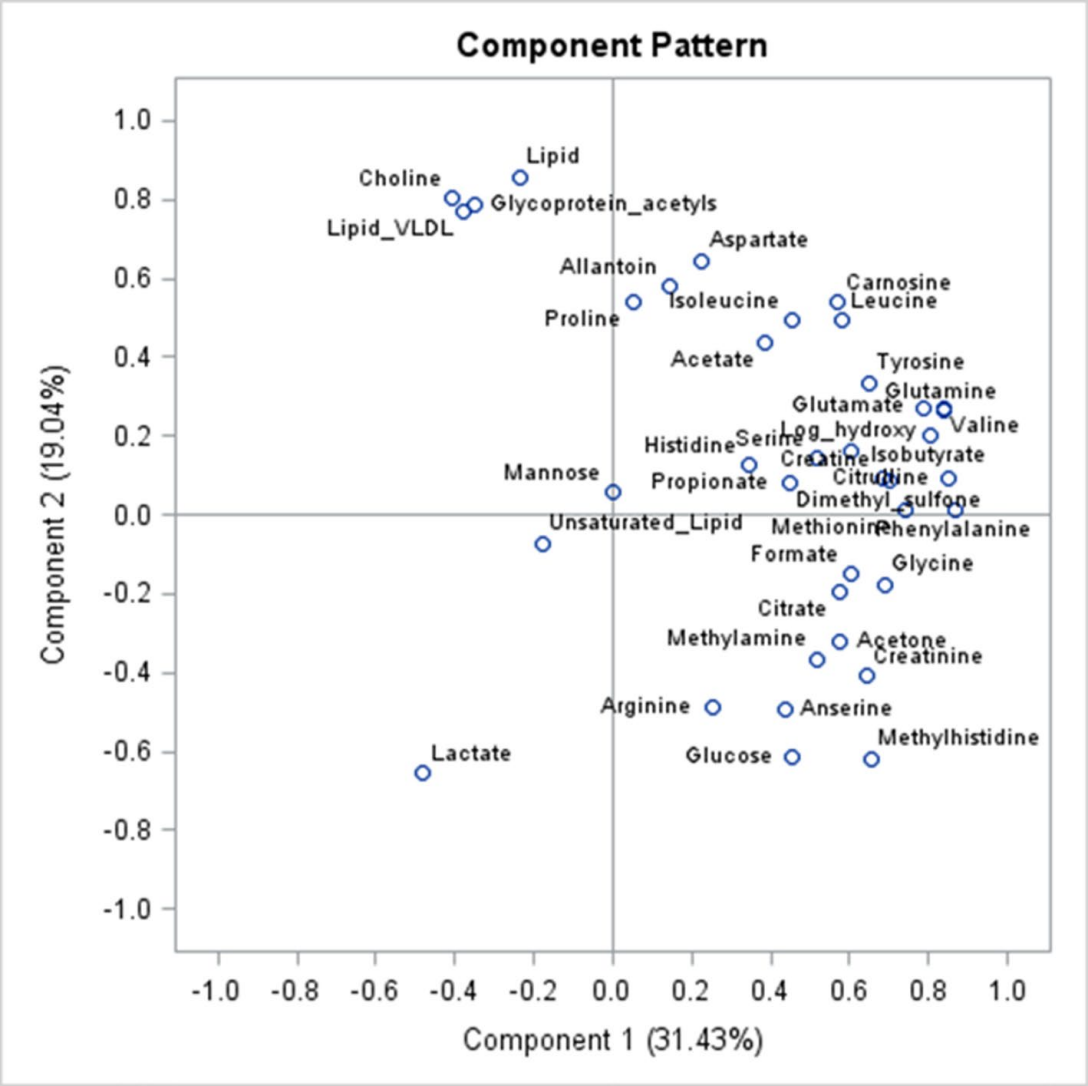
Loading plot for principal components 1 and 2 with 38 metabolites identified in blood of Wagyu crossbred steers.

**Figure 5:**
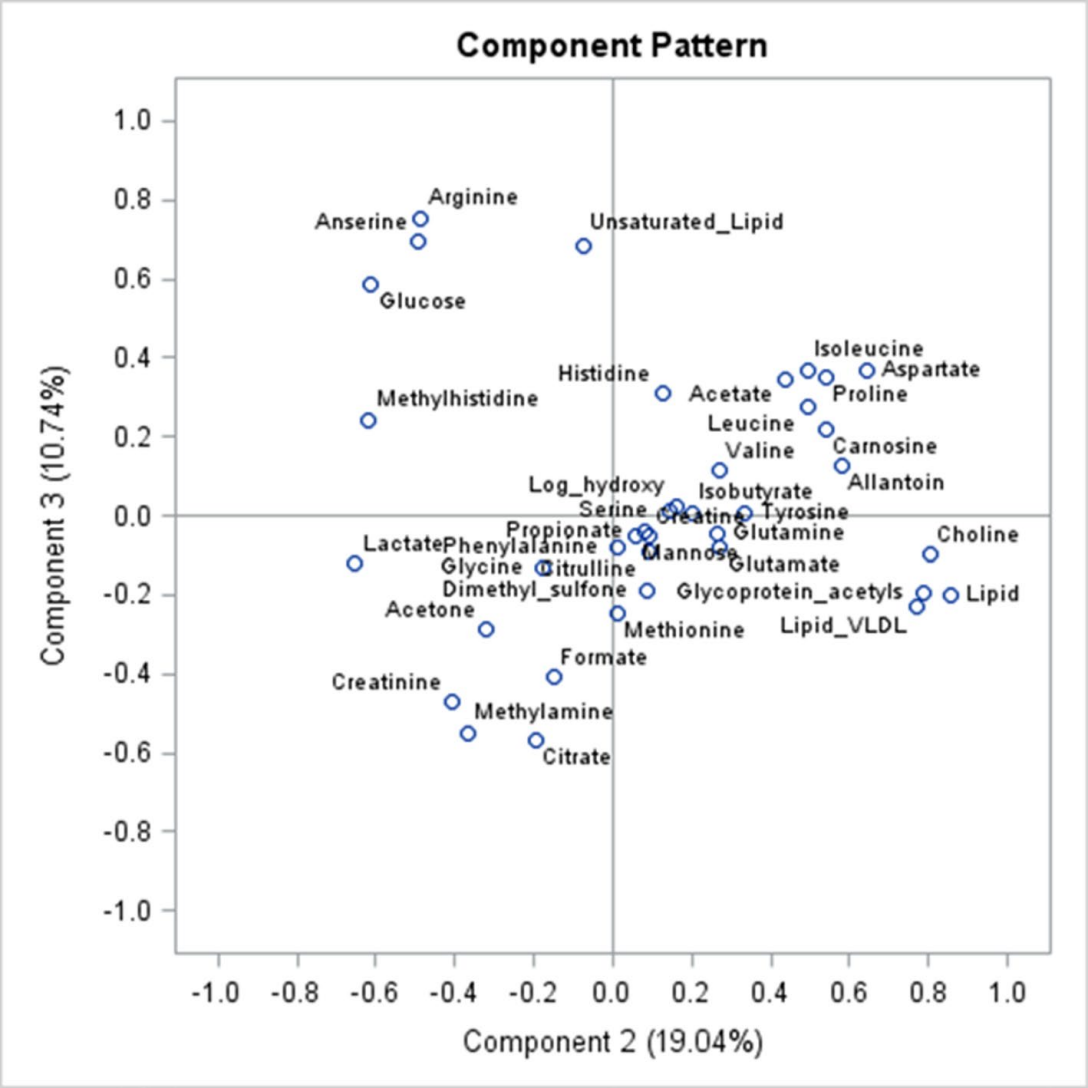
Loading plot for principal components 2 and 3 of 38 blood metabolites of Wagyu crossbred steers sampled at days 196 and 432 in a feedlot.

Figure 4 shows the loading or pattern plot for PC1 and PC2 which explained approximately half of the variability in the dataset. Lipid groups (lipids, very low-density lipoprotein (VLDL), and glycoprotein acetyls) and choline showed negative loading on PC1 and positive loading on PC2 which characterised samples at day 196 as shown in Table 3. Metabolites with positive loading on PC1 and negative loading on PC2 were glucose, methyl histidine, arginine, anserine and creatinine (Fig. 4) which characterised samples at day 432. Only lactate and the lipid groups showed negative loading on PC1 (Fig. 4). Allantoin, acetate and amino acids (aspartate, leucine, isoleucine, carnosine, and proline) showed high positive loadings on both PC1 and PC2 (Fig. 4). Figure 5 shows the loading plot for PC2 and PC3 where approximately 30% of the variation was explained and the lipids (VLDL, glycoprotein acetyls and lipids) and choline clustered together with high positive loading on PC2 and negative on PC3. Arginine, anserine, unsaturated lipids, and glucose had high positive loading on PC3, whereas creatinine, citrate and methylamine showed high negative loading (Fig. 5).

### Metabolite relative concentrations and relationships with marbling

The average relative concentration (RC) of each metabolite for each sampling point and the regression coefficient between the RC and marbling are shown in Table 4. The CM × time interaction was significant (*P* > 0.05) for 9 metabolites (3-hydroxybutyrate, acetate, allantoin, histidine, isobutyrate, methyl histidine, phenylalanine, tyrosine, and valine). Only 5 of 38 identified metabolites were not affected by time-point (*P* > 0.05); these were: arginine, mannose, methyl histidine, propionate, and serine (Table 4). Of those metabolites with no significant CM × time interaction, fifteen metabolites showed an increase (*P* < 0.05) in RC from day 196 to day 432; these were: acetone, anserine, citrate, citrulline, creatine, creatinine, dimethyl-sulphone, formate, glucose, glutamate, glutamine, glycine, lactate, methionine, and methylamine. In contrast, ten metabolites showed a decrease (*P* < 0.05) in RC from day 196 to day 432; these were aspartate, carnosine, choline, glycoprotein acetyls, isoleucine, leucine, VLDL, lipids, proline, and unsaturated lipids. The average RC did not differ (*P* < 0.05; Table 4) between days 196 and 432 for 3-hydroxybutyrate, tyrosine and valine; however, both the intercept and slope differed between time-points.

**Table 4:** The effect of days in a feedlot (time) on the relative concentration of blood metabolites, and the relationship between metabolites and marbling (CM) in Wagyu crossbred steers.

Metabolite	Time		P-value	Marbling regression coefficient	CM P-value	CM × time P-value
	Day 196	Day 432				
3-Hydroxybutyrate _log_	4.92 ± 0.0132	4.94 ± 0.0132	< 0.001	0.0082 ± 0.2133	0.049	< 0.001
Acetate _log_	4.74 ± 0.025	4.48 ± 0.025	< 0.001	0.94 ± 0.429	0.418	0.016
Acetone	18.02 ± 0.236	22.38 ± 0.235	< 0.001	− 0.023 ± 0.034	0.497	0.723
Allantoin	23.37 ± 0.307	19.08 ± 0.307	< 0.001	− 0.054 ± 0.052	0.044	0.042
Anserine	262.9 ± 1.71	266.9 ± 1.71	0.024	0.93 ± 0.252	< 0.001	0.069
Arginine	655.1 ± 3.92	657.4 ± 3.91	0.575	1.80 ± 0.57	0.002	0.209
Aspartate	14.07 ± 0.18	11.52 ± 0.180	< 0.001	0.021 ± 0.024	0.378	0.522
Carnosine	27.11 ± 0.233	25.36 ± 0.232	< 0.001	0.0043 ± 0.035	0.898	0.540
Choline	455.9 ± 3.71	401.3 ± 3.70	< 0.001	− 2.09 ± 0.549	< 0.001	0.749
Citrate	139.9 ± 1.42	167.7 ± 1.42	< 0.001	0.47 ± 0.197	0.017	0.098
Citrulline	20.42 ± 0.175	21.34 ± 0.175	< 0.001	0.026 ± 0.026	0.333	0.099
Creatine	265.9 ± 2.66	272.3 ± 2.66	0.008	0.77 ± 0.407	0.061	0.288
Creatinine	36.31 ± 0.416	45.46 ± 0.416	< 0.001	0.012 ± 0.062	0.850	0.581
Dimethyl sulfone	24.49 ± 0.232	25.99 ± 0.231	< 0.001	− 0.025 ± 0.034	0.465	0.858
Formate	3.89 ± 0.059	4.92 ± 0.059	< 0.001	− 0.016 ± 0.008	0.045	0.116
Glucose	1613.5 ± 10.63	1677.9 ± 10.60	< 0.001	5.71 ± 1.573	< 0.001	0.073
Glutamate	21.0 ± 0.196	21.7 ± 0.20	< 0.001	− 0.016 ± 0.029	0.591	0.273
Glutamine	251.6 ± 1.93	256.9 ± 1.92	0.002	− 0.11 ± 0.296	0.712	0.098
Glycine	116.9 ± 1.28	130.8 ± 1.28	< 0.001	0.0656 ± 0.186	0.727	0.402
Glycoprotein acetyls	256.4 ± 1.80	236.7 ± 1.79	< 0.001	− 1.04 ± 0.268	< 0.001	0.110
Histidine	286.5 ± 2.55	273.0 ± 2.53	< 0.001	0.92 ± 0.428	0.382	0.007
Isobutyrate	113.9 ± 0.99	115.4 ± 0.98	0.012	0.43 ± 0.166	0.185	0.003
Isoleucine	184.8 ± 1.16	173.8 ± 1.16	< 0.001	0.007 ± 0.162	0.965	0.286
Lactate	723.7 ± 17.79	830.8 ± 17.69	< 0.001	2.23 ± 2.559	0.388	0.364
Leucine	156.6 ± 1.25	148.1 ± 1.245	< 0.001	0.09 ± 0.18	0.622	0.289
Lipid	1201.4 ± 6.36	1133.1 ± 6.34	< 0.001	− 3.82 ± 0.933	< 0.001	0.134
VLDL	807.7 ± 5.40	752.7 ± 5.36	< 0.001	− 3.53 ± 0.773	< 0.001	0.062
Mannose	4.67 ± 0.091	4.89 ± 0.091	0.089	− 0.021 ± 0.011	0.062	0.289
Methionine	224.9 ± 2.058	245.6 ± 2.06	< 0.001	0.09 ± 0.327	0.775	0.075
Methylamine	29.27 ± 0.493	41.05 ± 0.495	< 0.001	0.197 ± 0.069	0.005	0.099
Methyl histidine	194.5 ± 1.23	209.6 ± 1.22	0.695	0.61 ± 0.208	0.033	0.047
Phenylalanine	35.77 ± 0.297	38.04 ± 0.296	< 0.001	0.009 ± 0.044	0.897	0.054
Proline	37.81 ± 0.544	31.24 ± 0.543	< 0.001	− 0.024 ± 0.067	0.719	0.558
Propionate	17.08 ± 0.156	17.25 ± 0.156	0.352	0.08 ± 0.022	< 0.001	0.245
Serine	17.06 ± 0.231	17.07 ± 0.231	0.965	0.009 ± 0.029	0.754	0.542
Tyrosine	66.58 ± 0.656	67.52 ± 0.654	0.026	0.057 ± 0.111	0.273	0.010
Unsaturated lipid	440.1 ± 1.79	428.6 ± 1.78	< 0.001	0.21 ± 0.252	0.401	0.365
Valine	275.4 ± 2.13	275.8 ± 2.12	0.032	0.73 ± 0.36	0.322	0.026

*VLDL* very low-density lipoprotein.

There were 11 metabolites with significant (*P* < 0.05; Table 4) regression coefficient between marbling and RC as indicated by the main effect of marbling with non-significant interaction. These included 5 metabolites (choline, formate, glycoprotein-acetyls, VLDL and lipids) that had a negative correlation with CM (*P* < 0.05). Six metabolites had a positive correlation with CM (*P* < 0.05; Table 4) including anserine, arginine, citrate, glucose, methylamine, and propionate). In addition, creatine showed a tendency for a positive relationship with marbling (*P* = 0.06).

The results for the metabolites with significant CM × time interactions are shown in Table 5 through the regression coefficient for each time-point. Allantoin and tyrosine showed a negative association (*P* < 0.05) with marbling at day 196 but no association at day 432 (P > 0.05). The metabolites 3-Hydroxybutyrate, acetate, histidine, isobutyrate, methyl histidine and valine showed a positive association (P < 0.05) with marbling at day 432 however there was no apparent association at day 196 (Table 5).

**Table 5:** Regression coefficients of the relative concentration of blood metabolites against marbling for metabolites with a significant interaction between marbling and days in a feedlot for Wagyu crossbred steers.

Metabolite	Days in a feedlot			
	Day 196		Day 432	
	Reg. Coeff ± SE	P-value	Reg. Coeff ± SE	P-value
3-Hydroxybutyrate _log_	− 0.002 ± 0.002	0.545	0.008 ± 0.002	< 0.001
Acetate _log_	− 0.004 ± 0.004	0.384	0.009 ± 0.004	0.030
Allantoin	− 0.142 ± 0.053	0.008	− 0.054 ± 0.052	0.298
Histidine	− 0.022 ± 0.458	0.962	0.920 ± 0.448	0.041
Isobutyrate	− 0.031 ± 0.170	0.855	0.429 ± 0.167	0.011
Phenylalanine	− 0.010 ± 0.052	0.842	0.055 ± 0.051	0.282
Methyl histidine	0.156 ± 0.213	0.465	0.608 ± 0.208	0.004
Tyrosine	− 0.255 ± 0.1138	0.026	0.055 ± 0.1109	0.622
Valine	− 0.114 ± 0.3695	0.758	0.746 ± 0.3614	0.040

*Reg. Coeff.* regression coefficient, *SE* standard error.

## Discussion

The first aim of the present study was to compare the metabolome for individual Wagyu crossbred steers at days 196 and 432 in a feedlot. These time-points were 236 days apart, and 272 and 36 days before animals were slaughtered and carcass traits measured. Previous studies sampling cattle at different times for metabolomic analysis had shorter time intervals and were undertaken in younger animals earlier in the feedlotting process^7,16–18^. Two important differential features of the present study were (1) the long interval between the two sampling points and (2) the sampling of older animals that were presumed to be undergoing a greater rate of intramuscular fat (IMF) accretion rate as a result of greater physiological maturity. The relative concentrations for 33 out of 38 metabolites were different between day 196 and 432. These findings indicated that the blood metabolome in steers changes with time in a feedlot. These changes could be due to a number of factors including age, body maturation (e.g. rate of IMF accretion), prevailing environment, and diet. Yang et al.^19^ reported differences in the relative abundance of 56 plasma metabolites between steers fed a diet with low corn grain (29% of DM) and those fed a diet with high corn grain (49% of DM). The changes in diet from day 196 to 432 in the present study were minor, with the diet fed at day 432 having 3.85% lower NDF and 6.0% lower forage. Furthermore, laboratory analysis of diets 3 (196 days sampling) and 4 (432 days sampling) indicated no significant differences in the chemical composition. The concentration of crude fat of both diets was of interest because it suggests that the availability of lipids was similar at both sampling times. Therefore, the changes in the RC of metabolites and lipid groups, and the relationships presented between metabolites and marbling reflect changes in animal metabolism rather than changes in the diet consumed. The average ambient temperature in the sub-tropical climate of the present study also showed only a minor difference between time-points (day 196 was 34 °C; day 432 was 30 °C). Therefore, the changes in the plasma metabolome between days 196 and 432 were presumed to be due to age, body maturation, and metabolic and physiological status.

The second aim of the present study was to determine the relationships between the metabolome and marbling, and the effect of days on feed (time points) on these relationships. Fourteen metabolites which were associated with marbling had correlation coefficients of 0.35–0.60 between days 196 and 432 (3-hydroxybutyrate, propionate, glucose, choline, anserine, arginine, citrate, methylamine, methyl histidine, and lipid groups—lipids, VLDL and glycoprotein-acetyls). This finding indicated that the ranking of individual animals based on the RC of these metabolites was consistent from days 196 to 432. In addition to this, the fact that many of these metabolites were associated with marbling encourage potential applications of metabolomics to aid in selecting animals for propensity to marble. Other metabolites (creatine, allantoin, glutamine, and methionine) had relatively high correlation coefficients (r > 0.60) between days 196 and 432; however, none of these metabolites were correlated with marbling in the present study except for a positive trend for creatine. Arginine, citrate, glucose, and propionate were associated with marbling as the main factor. Propionate enters the TCA cycle and is converted to glucose in the liver (gluconeogenic pathway), and glucose is then used for fatty acid synthesis to be finally used for IMF deposition^2^. Furthermore, arginine and citrate can also be converted to acetyl-coA in the TCA cycle and later used for fatty acid synthesis^20^. The positive relationship between propionate and marbling of the present study was also observed in an earlier study with Wagyu crossbred steers with a similar genetic background^7^. Similar to the present study, the earlier study^7^ also reported positive associations between marbling and the RC of creatine, 3-hydroxybutyrate, acetate, histidine, isobutyrate, and valine. The similarities between the two studies could be explained by the similar genetic background and comparable management and feeding systems. In addition, the consistency of these relationships between metabolites and marbling may represent common critical metabolic pathways involved in IMF synthesis and deposition. However, more metabolites were associated with marbling in the present study compared to the previous sampling younger animals^7^. In addition, more metabolites were associated with marbling at 432 compared to 196 days (3-hydroxybutyrate, acetate, histidine, isobutyrate, methyl histidine, and valine). These results suggest that the association between the metabolome and marbling increased with age or degree of maturity. However, further studies are required to confirm this hypothesis.

Glucose showed a positive association with marbling independently of sampling time however there was a tendency for a marbling × days on feed (DOF) interaction (P = 0.07) because the slope of the regression coefficient tended to be greater at day 196 compared to day 432 (data not shown). In the earlier study with Wagyu crossbred steers, glucose showed a linear decrease with marbling at day 65, no association at day 119, and positive association at day 163 in the feedlot^7^. The findings from the present and earlier studies could be interpreted to suggest that the relationship between glucose and marbling becomes stronger and more positive as steers mature and glucose demand for marbling increases. It has been reported that dairy cows can respond to increased glucose demand during post-partum by doubling liver gluconeogenesis^21^. Therefore, it is plausible that animals with higher propensity to marble have faster gluconeogenic rate producing glucose needed for de novo synthesis of both fatty acids and triglycerides as previously reviewed^1,2^.

Similarly, there was a negative association between VLDL and marbling in the present study with a tendency for a marbling × time interaction (P = 0.06) because the regression coefficient (slope) was lesser at day 196 compared to day 432 (data not shown). In the earlier study with Wagyu, the association between lipids and marbling was positive at day 65, not significant at day 119, and negative at day 163^7^. In both studies, therefore, the negative association between lipids and marbling became stronger in older animals (i.e. more negative). It is possible that the greater rate of IMF accretion as steers mature is achieved, in part, by a more rapid uptake of circulating lipids for marbling or IMF deposition, which results in lesser blood concentrations. In contrast, higher marbling seems to be ‘fuelled’ by higher concentration of glucose and its precursor propionate in blood.

Five metabolites (3-hydroxybutyrate, histidine, isobutyrate, methyl histidine and valine) showed a positive linear relationship with marbling at day 432 but not day 196. In contrast, the metabolites allantoin and tyrosine showed a negative association with marbling at day 196 but not day 432. These results further illustrate the complexity of relationships between blood metabolites and marbling and as noted above, further studies are needed to gain a deeper understanding on the metabolome and marbling in cattle throughout different stages of maturity.

The principal component analysis (PCA) and linear models revealed metabolic patterns related to stage of physiological maturity and the relationships with the metabolism of IMF. The PCA score plot indicated that PC2 and PC3 accounted for a lower proportion of the variance of the dataset compared to PC1. However, PC2 and PC3 seemed more suitable to differentiate between DOF based on the proportion of the variance explained in the GLM models. Furthermore, PC2 and PC3 showed the highest negative and positive correlation with marbling, respectively. Therefore, PC2 and PC3 seem to explain stage of maturity and IMF deposition better compared to the rest of the PC’s. Positive values for both PC2 and PC3 were found in animals sampled at 196 DOF, and negative values for both PC2 and PC3 at 432 DOF. The loading plots highlighted those metabolites with positive loading on PC2 including choline, lipid groups (lipids, VLDL and glycoprotein acetyls), acetate, allantoin, and a group of amino acids (aspartate, proline, isoleucine, leucine, and carnosine). Furthermore, these metabolites showed a significant decrease of the RC from 196 to 432 DOF and choline, lipids and VLDL were also negatively associated with marbling in the present experiment. Therefore, maturity seems to be associated with a reduction in the circulating concentration of metabolites involved in lipid metabolism. Lipids are the primary components of fat tissue in animal bodies whereas choline is a precursor for the synthesis of hepatic VLDL formed by choline phospholipids^22,23^. In post-partum dairy cows, choline plays an important role in the export of triacylglycerol from the liver promoting phosphatidylcholine synthesis in the Kennedy pathway to improve coping with negative energy balance and increase milk production^22,24^. Therefore, this group of metabolites seem to be important during maturity and fat deposition showing lower concentration with higher maturity when IMF deposition is expected to be faster. In addition, animals with higher ability to marble show lower concentration of metabolites involved in lipid metabolism. It is plausible that this is a result of faster uptake of circulating lipids by body tissues required for fat deposition.

In contrast to lipid groups, glucose, anserine, and arginine had high negative loading on PC2 and positive on PC3. The RC of these metabolites were positively associated with marbling and increased with DOF. Therefore, it seems plausible that these metabolites act as metabolic fuels for lipid synthesis which are then used for fat deposition as the animal matures with age. Thus, animals with higher availability of these metabolic fuels may favour lipid synthesis and fat deposition, and these animals seem to uptake circulating lipids at a faster rate clearing them from the bloodstream. Furthermore, there were 15 metabolites that increased from 196 to 432 DOF, some of these metabolites included molecules that are important in glycolysis to produce pyruvate and ATP including creatine, glucose, glutamate, glycine, lactate, methionine and phenylalanine^25^. Pyruvate then enters the TCA cycle, as it is glutamate which is involved with the α-Ketoglutarate section of the TCA. Methionine can be converted to succinyl-CoA and then used for glycogenesis whereas phenylalanine can enter the fumarate part of the TCA^25^. These findings may suggest that the ability of animals to produce energy via both glycolysis and TCA increases with age or maturity, and this may be linked to increased synthesis and uptake of lipids.

## Conclusions

Significant changes in the relative concentration of metabolites and of the metabolic profile occurred in crossbred Wagyu steers sampled at two distinct time points (early and late) in the feedlotting process. These changes demonstrate the importance of stage of maturity on metabolic processes and are likely related to fat metabolism and deposition, at least partially. Maturity is accompanied by an increase in the relative concentration of metabolites that participate in metabolic pathways for energy production and precursors used for fat acid synthesis such as citrate, creatine, creatinine, formate, glucose, glutamate, glycine, lactate, and methionine. In contrast, the concentration of circulating metabolites related to lipid metabolism and fat deposition decrease with stage of maturity such as choline, lipids, and acetyl groups. Several amino acids involved in protein metabolism also decreased with time including proline, leucine, isoleucine, histidine, carnosine, allantoin and aspartate. Further to this, Wagyu steers with higher marbling at the time of slaughter tend to show greater concentrations of propionate, 3-hydroxybutyrate, acetate, creatine, glucose, anserine, and arginine but lower blood concentration of lipid groups, choline and acetyl groups. Sampling time in relation to stage of maturity needs to be considered to understand results from metabolic studies and for practical applications including the prediction of valuable carcass traits such as marbling.

## Data Availability

The data and computing programs used in this manuscript may be available from the corresponding author on request and if approved by funding bodies to do so. Restrictions apply to the availability of these data, which were used under license for the current study, and so are not publicly available.
